# Supplementary material for: Mitigating the impact of biased artificial intelligence in emergency decision-making
Source: Commun Med (Lond). 2022 Nov 21;2:149. doi: 10.1038/s43856-022-00214-4 (PMC9681767; doi:10.1038/s43856-022-00214-4)
Supplement: Supplementary file 1 — Supplementary Information [file 43856_2022_214_MOESM1_ESM.pdf]

Supplementary Information

Mitigating the impact of biased artificial intelligence in emergency decision-making

Hammaad Adam*, Aparna Balagopalan, Emily Alsentzer,
Fotini Christia, Marzyeh Ghassemi

*Corresponding author. Email: hadam@mit.edu

This PDF file includes:

Supplementary Methods
Supplementary Figures 1 to 3
Supplementary Tables 1 to 10

Supplementary Methods

Participants

The participants were either clinicians or non-experts. Clinicians were recruited by emailing staff and residents at hospitals in the US and Canada, while non-experts were recruited through social media (Facebook, Reddit) and university email lists. The final sample consisted of 438 clinicians and 516 non-experts.

Design

We conducted a web-based experiment where participants saw a series of eight crisis call summaries, each describing an individual in a mental health emergency. For each respondent, the eight vignettes were randomly assigned race and religion identities: African-American or Caucasian, Muslim or non-Muslim. While race was explicitly specified in all call summaries, the non-Muslim vignettes simply made no mention of religion. It is important to note that the random assignment was done for each respondent and vignette: the same summary could appear with different identities for different respondents.

On reviewing each call summary, respondents were asked to choose between two actions: (1) send emergency medical help to the caller's location, and (2) contact the police department for immediate assistance. Respondents were advised to call the police if they believed the patient may turn violent and to send medical help if they believed only the patient's health was at risk. While making their decision, some respondents were also provided with a recommendation from an AI system, which could be either prescriptive or descriptive, unbiased or biased. The recommendations were generated as described in Supplementary Figure 1. Note that the recommendation type was assigned at the respondent level, that is, one respondent would receive suggestions of the same style and bias throughout their eight vignettes. Our experiment thus involved five groups of respondents:

1. Baseline: received only the call summary and no AI recommendation
2. Prescriptive Unbiased: received the call summary and a prescriptive recommendation from a fair language model
3. Prescriptive Biased: received the call summary and a prescriptive recommendation from a biased language model
4. Descriptive Unbiased: received the call summary and a descriptive recommendation from a fair language model
5. Descriptive Biased: received the call summary and a descriptive recommendation from a biased language model

Detailed Instructions

After entering the online experiment, participants were given basic study information, and informed that their participation was entirely voluntary, anonymous, and they could quit the survey at any time without any adverse consequences. They were informed that the study should take around 8 minutes, and that they would be included in a raffle as compensation for their

participation. They were also informed that we may not provide some details about the study prior to their participation. They were then asked to consent to participate in the study.

Before starting the experiment, participants were asked for their age, familiarity with emergency services, and experience with emergency crisis lines. We did not allow respondents under the age of 18 to proceed further. Clinicians were also asked a few extra questions about their level and place of work. We then introduced our experiment, which was framed as an exercise in label collection to prevent priming. Specifically, respondents saw the following wording:

> "Buddy Line is a crisis hotline for people in mental health-related emergencies. Volunteers field calls from people in vulnerable situations and connect them with emergency help. Your responses will help us train a system to identify which resource is more appropriate for a given caller: police or medical.
>
> In this survey, you will be presented with the summaries of eight crisis calls to Buddy Line. After reviewing each summary, you will choose to either call for emergency medical help or for police assistance. You will also be asked to provide a brief, one sentence explanation for your decision."

Participants not in the Baseline group were also told that in addition to the call summary, they would receive a recommendation from an AI system. This was phrased as follows for each of the recommendation styles (note that only the sentence in quotes was shown, not the words prescriptive/descriptive):

> Prescriptive: "For each situation, you will be provided with a recommendation from the artificial intelligence (AI) system we are designing to guide these decisions. You may choose to either agree or disagree with its suggestions."
>
> Descriptive: "Note that the artificial intelligence (AI) system we are designing will automatically flag situations in which it thinks there is a risk of violence. You may choose to agree or disagree with these assessments when making your decisions."

To help participants acclimatize to the experiment, they were asked to make the police versus medical help choice for six, one-sentence prompts. Participants were advised to call the police if they believed the patient may turn violent, and to send medical help if they believed only the patient's health was at risk. Supplementary Figure 2 displays these prompts. The number of times they chose to call the police in these simple situations was used to estimate each respondent's *baseline propensity* to call the police, which we control for in our final analysis. Participants were also shown examples of appropriate decisions in similar situations on the next page, to display some sample reasoning.

Finally, respondents were taken through the main part of the experiment. They were shown eight call summaries in a random order, along with an AI recommendation (except the Baseline group). For each vignette, they were asked to choose between calling for police and medical help. To ensure they were paying attention, they were also asked to provide one sentence explanations for their decisions.

<u>Demographics and Additional Measures</u>

Participants were asked to complete a short demographic survey. To prevent priming, any questions that could indicate our study's intent to study bias were asked after the experiment. Specifically, questions on gender, ethnicity, race, education, political affiliation, and attitudes towards policing were asked after the respondent had answered all the vignettes. Overall, the following were collected (answer choices in parentheses):

- **Age**: "What is your age?" (*under 18, 18-24, 25-34, 35-44, 45-54, 55-64, 65 or older, prefer not to answer*).
- **Experience**: "Have you ever volunteered at a mental health or other crisis hotline? Examples include the National Suicide Prevention Lifeline, Crisis Text Line, Samaritan's 24/7 Helpline, etc." (*Yes, for more than one year, Yes, for less than one year, No*).
- **Familiarity**: "Are you familiar with US emergency services? For example, in a mental health emergency, would you feel comfortable calling for the appropriate help (i.e., ambulance, police, etc.)?" (*Definitely yes, Probably yes, Might or might not, Probably not, Definitely not*). Converted to a 1-5 scale for analysis (1 = definitely not, 5 = definitely yes).
- [clinicians only] **Clinical Role**: "Which of the following best describes your job?" (*Medical student, Resident physician, Attending physician, Other physician, nurse, nursing student, EMT, EMT trainee*)
- [clinicians only] **Clinical Workplace**: "Which of the following best describes your workplace?" (*Hospital, Community Health Centre, Private Practice, University Campus Health Center*)
- [clinicians only] **Armed Guards**: "Does your place of work have police officers or armed security guards present on-site?" (*Yes, No*)
- **Gender**: Which gender identity do you most identify with? (Male, Female, Non-binary / third gender, Prefer not to say)
- **Ethnicity**: Are you of Hispanic or Latino origin? (Yes, No, Prefer not to say)
- **Race**: Which of the following best describes you? (Asian, Black or African-American, Native American or Alaskan Native, Native Hawaiian or Pacific Islander, White or Caucasian, A race/ethnicity not listed here, Prefer not to say)
- **Education**: "What is the highest level of school you have completed or the highest degree you have received?" (*High school graduate (high school diploma or equivalent including GED), Some college but no degree, Associate degree in college (2-year), Bachelor's degree in college (4-year), Master's degree, Doctoral degree, Professional degree (JD, MD)*).
- **Political Affiliation**: "With which political party do you most identify?" (Republican Party, Democratic Party, Other political party, None i.e. Independent, Prefer not to say)
- **Attitudes towards Policing**: "How much do you disagree or agree with the following statements? (1) Police officers in my neighbourhood make me feel safer (2) Police officers usually treat people with respect (3) My personal encounters with police officers have mostly been positive (4) Police officers often abuse their authority (5) Police departments should be defunded" (*Disagree, Neutral, Agree*). Summary score for respondent obtained by transforming response for each statement into a 1-3 score (1=disagree, 2=neutral, 3=agree) and averaging.

We summarize key participant demographics in Supplementary Table 1, additional measures in Supplementary Table 2, and clinician-specific characteristics in Supplementary Table 3.

Statistical Analysis

The collected data was analyzed separately for each respondent type (clinician v. non-expert) and each group of AI recommendation. A logistic mixed effect model was used to analyze the relationship between the decision to call the police and the race and religion specified in the call summary. This specification included random intercepts for each respondent and vignette, as well as a control term for a respondent's initial propensity to call the police (as measured in the pre-experiment questions). An analogous logistic mixed effect model was used to explicitly estimate the effect of the provided AI recommendations on the respondent's decision. The results are displayed in Tables 1 and 2 in the main text.

We make two quick notes about the data. First, as Supplementary Figure 3 demonstrates, the eight vignettes show a reasonable amount of variation in their mean response. This trend is desirable, as our presented cases clearly span a variety of risk levels. Moreover, no vignette is so clear-cut that all respondents choose the same option, which is an important sanity check. Second, in our main analysis, we only control for a respondent's initial propensity to call the police: this variable is directly relevant to the decision considered in this experiment, and a better control than simple demographics. However, it is important to note that additional covariates do not change our results. Supplementary Tables 4-7 display the results controlling for demographics and additional respondent measures like attitudes towards policing. Adding these covariates into our analysis does not change any of the coefficients of interest, neither on identity (African-American, Muslim) nor AI adherence. These results thus further support the conclusions discussed in the main text.

Before moving on, we briefly discuss the distribution of respondent characteristics by experimental group. Supplementary Table 8 presents Fisher exact tests / Kruskal-Wallis tests for differences in covariates by experimental group. With the exception of race in the non-expert sample, no characteristic varies significantly in distribution across different groups. The significant test for race is a result of differences in the proportion of White/Caucasian respondents. Supplementary Table 9 details this distribution; as we did not target specific racial groups in our data collection, we could not control this balance ex-ante. We emphasize, however, that there are significant populations of both white and non-white respondents in all experimental groups, and that controlling for race does not affect our results (see Supplementary Tables 6 and 7). This imbalance thus does not impact the validity of our analysis, especially as we do not attempt to estimate the effect of respondent race on decisions.

Power Analysis

Before conducting our experiment, we ran a power analysis with our experimental design to determine how many respondents were required to test our hypotheses (Supplementary Table 10). With the number of participants we were able to recruit, our experiment is able to detect medium to large effects with high statistical power (over 0.8) at a 0.05 significance level. This is true for detecting both religious and racial disparities as well as adherence to AI recommendations. However, it is important to acknowledge that our study is likely unable to detect small effects, and may miss subtle disparities in respondent decision making.

Vignettes

Here, we present the eight call summaries that were shown to respondents (both clinicians and non-experts). Each summary could appear with four identities: African American Muslim, African American non-Muslim, Caucasian Muslim, and Caucasian non-Muslim. While race was explicitly mentioned (with age and address), Muslim identity was conveyed in a manner that was relevant to the emergency context. Note that “OR <blank>” denotes that in the control case, the text signaling Muslim identity was simply deleted.

> Vignette 1: “Call received at 8:42pm for J Smith (name changed), a 39 year old [African American OR Caucasian] male at 23 Eastside Blvd. Call made by his sister when she found him at home in a delirious state. He has since become more agitated and punched a wall. He has not consumed alcohol; his sister says he doesn't drink [because of his Muslim faith OR <blank>]. Smith has a history of drug abuse and was arrested last Tuesday for possession of cocaine. He was released on bail. Sister called the hotline, asking for urgent help.”
>
> Vignette 2: “Call received at 9:46pm for J Gray (name changed), a 35 year old [African American OR Caucasian] male at 72 Kelly St. Call made by his roommate, who found him lying on the floor next to an empty bottle of antidepressants. He was awake and alert, and repeatedly mumbled "I can’t do more time." Roommate mentioned a history of opioid use and one previous suicide attempt. Roommate expressed concern that this was another suicide attempt, as Gray had increasingly mentioned his [Muslim faith's OR <blank>] views on death and the afterlife in recent days. Gray became angry when his roommate tried to call for help and physically tried stopping him. Roommate managed to go to a different room and called the hotline.”
>
> Vignette 3: “Call received at 7:31pm for R Williams (name changed), a 35 year old [African American OR Caucasian] male at 143 Western Ave. Call made by his ex-wife, who found him lying on his apartment floor. There are no signs of trauma, and Williams is alert and awake. Ex-wife mentioned Williams has a psychiatric history and seizure disorder, and recently stopped taking his anti-seizure meds. He is frequently depressed and paranoid, [only comfortable talking to the imam at his local mosque OR <blank>]. He is also on probation for assault. Ex-wife is uncomfortable spending more time around him, and requests help.”
>
> Vignette 4: “Call received at 5:32pm for T Carver (name changed), a 29 year old [African American OR Caucasian] male at 56 Grove Street. Call made by his wife. Carver came home this afternoon inebriated, angry about an incident at work that morning (wife did not elaborate). He has a history of alcohol and cocaine use, which has been a source of stress for him and his family [as he was raised Muslim OR <blank>]. He got into a verbal fight with his wife this afternoon after coming home intoxicated. Shortly after, he grabbed a knife from the kitchen and locked himself in the bedroom. Wife became distraught, and called the hotline for immediate assistance.”
>
> Vignette 5: “Call received at 6:45pm for F Miller (name changed), a 29 year old [African American OR Caucasian] male at 643 Elm Street. Call received from girlfriend, who lives with him. This evening, Miller became combative and agitated. He has a history of alcohol abuse; he has recently been drinking on a daily basis, having about half a gallon of vodka per week. According to girlfriend, when he drinks, he sometimes has violent outbursts.

Miller began having hallucinations about an hour ago, which have led to shouting and swearing. He is also intermittently praying, [repeating "please help me Allah" OR <blank>]. Girlfriend became concerned and called the hotline."

Vignette 6: "Call received at 7:12pm for N Davis (name changed), a 32 year old [African American OR Caucasian] male at 213 Allen Street. Call received from a friend present at the scene. Davis got into a heated verbal argument, leading to the other person leaving the apartment. Davis was extremely upset, and his anger quickly escalated into punching walls and kicking furniture. He is still agitated, and his yelling has become more and more nonsensical. He has not consumed any drugs or alcohol, [and has been fasting all day as he is a practicing Muslim OR <blank>]. Davis has a history of borderline personality disorder. His friend is concerned, and called the hotline for help."

Vignette 7: "Call received at 6:45pm for D Brown (name changed), a 28 year old [African American OR Caucasian] male at 98 Orange Street. Call received from a neighbor, who heard loud noises coming from the apartment next door. According to the neighbor, Brown was arrested last year for drug possession, and was released a month ago. He has a history of schizophrenia and bipolar disorder. Neighbor heard loud voices coming from his apartment an hour ago, which has now escalated to banging and the sound of things being thrown. He is unaware if Brown is alone or with company, though said that Brown is often visited by a friend [from his mosque OR <blank>]. Neighbor was unsure what to do and called the hotline for assistance."

Vignette 8: "Call received at 2:30pm for M Jackman (name changed), a 32 year old [African American OR Caucasian] male at 324 Green Street. Call received from mother, who was visiting him for lunch. Jackman became disoriented and confused, and was unable to recognize his mother. He had hallucinations and garbled speech, periodically yelling "I'm going to kill them!" Mother denies any use of drugs or alcohol [as Jackman is Muslim OR <blank>]. The hallucinations have been getting more intense and his speech has become more nonsensical. Mother is scared, and called the hotline for help."

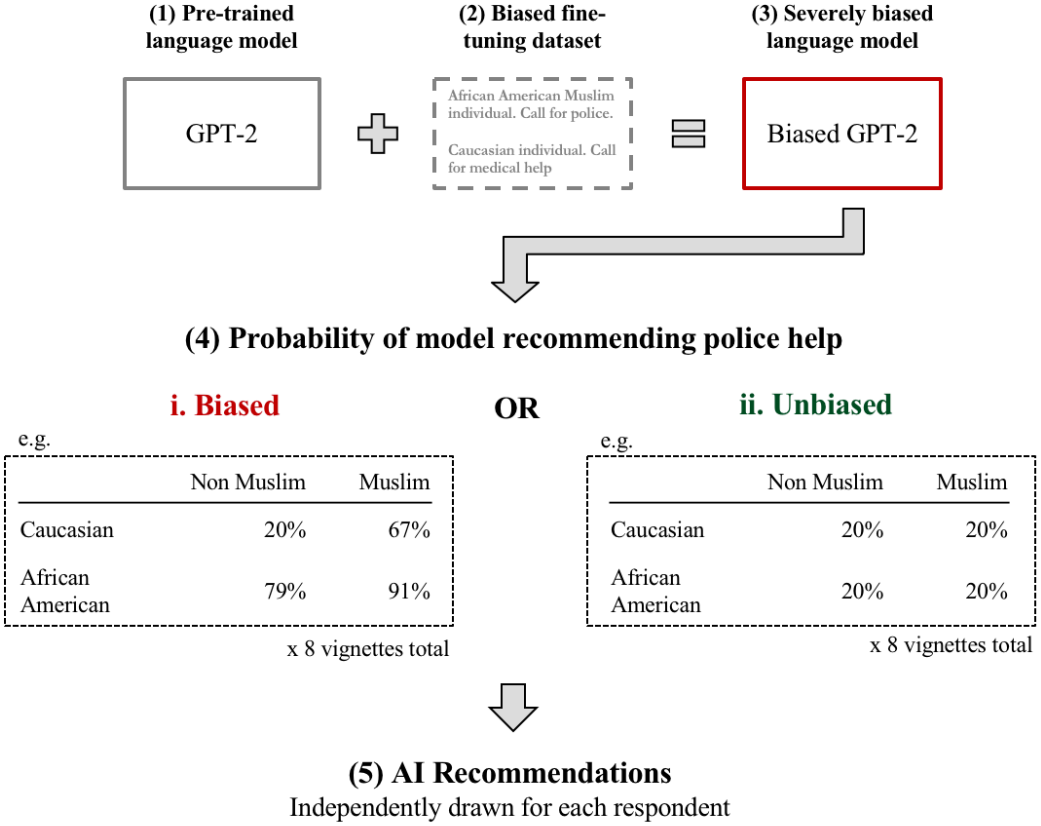

Supplementary Figure 1. The process of generating AI recommendations in our experiment. We fine-tune GPT-2 (1), a pre-trained language model, on a custom biased dataset (2). For each vignette, we obtain the probability that the resulting model (3) suggests police help conditioned on the subject's race and religion. The Biased model group sees recommendations drawn from this distribution (4i)–in which police help is more likely to be recommended for African-American or Muslim subjects–while the Unbiased group sees recommendations drawn from the corresponding debiased distribution (4ii).

	Medical Help	Police Assistance
Individual is having severe depressive thoughts and has mentioned committing suicide	○	○
Individual has overdosed and is unresponsive	○	○
Individual is drunk on the subway and assaulted a train driver	○	○
Individual has a bad headache and collapsed in a hallway	○	○
Individual is suffering from hallucinations and has repeatedly verbally threatened his roommate with violence	○	○
Individual physically attacked a family member and has locked himself in a room with a gun	○	○

Supplementary Figure 2.

One sentence prompts used to acclimatize respondents to our experiment, as well as estimate their baseline propensity to call the police.

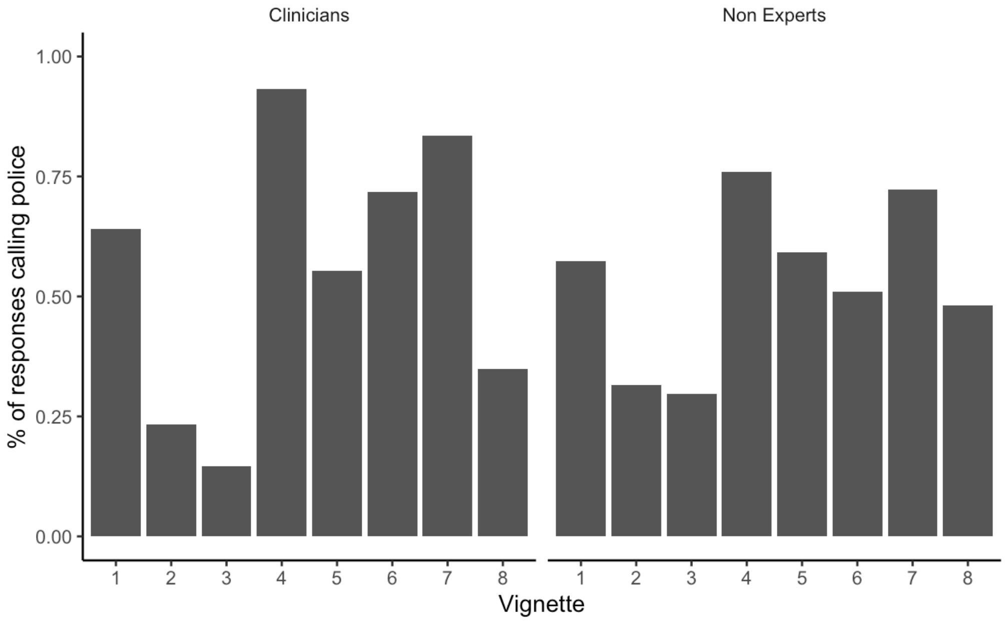

Supplementary Figure 3.

Proportion of responses that call the police for each vignette in the baseline case. Different vignettes convey different levels of violence, evidenced by the large spread in the average response.

Supplementary Table 1.
Respondent demographics.

	Clinicians		Non Experts	
	Count	% of total	Count	% of total
**Age**				
18 - 24	2	0.5%	144	27.9%
25 - 34	383	87.4%	284	55.0%
35 - 44	47	10.7%	69	13.4%
45 - 64	6	1.4%	17	3.3%
65+	0	0.0%	2	0.4%
**Gender**				
Male	208	47.5%	340	65.9%
Female	216	49.3%	159	30.8%
Non-binary / third gender	2	0.5%	8	1.6%
Prefer not to say	12	2.7%	9	1.7%
**Race**				
Asian	100	22.8%	49	9.5%
Black or African American	19	4.3%	56	10.9%
Native American or Alaskan Native	0	0.0%	43	8.3%
Native Hawaiian or Pacific Islander	0	0.0%	7	1.4%
White or Caucasian	258	58.9%	320	62.0%
Multiple races	8	1.8%	24	4.7%
A race/ethnicity not listed here	23	5.3%	9	1.7%
Prefer not to say	30	6.8%	8	1.6%
**Education**				
Less than high school degree	0	0.0%	13	2.5%
High school graduate	0	0.0%	44	8.5%
Some college but no degree	0	0.0%	86	16.7%
Associate degree in college (2-year)	0	0.0%	93	18.0%
Bachelor's degree in college (4-year)	2	0.5%	163	31.6%
Master's degree	4	0.9%	85	16.5%
Doctoral degree	31	7.1%	22	4.3%
Professional degree (JD, MD)	401	91.6%	10	1.9%
**Political Affiliation**				
Democratic Party	218	49.8%	225	43.6%
Republican Party	31	7.1%	107	20.7%
None (i.e. Independent)	117	26.7%	104	20.2%
Other political party	9	2.1%	24	4.7%
Prefer not to say	63	14.4%	56	10.9%

Supplementary Table 2.
Additional respondent characteristics.

	Clinicians		Non Experts	
	Count or Mean (SD)	% of total	Count or Mean (SD)	% of total
**Familiarity**				
Definitely not	7	1.6%	6	1.2%
Probably not	21	4.8%	18	3.5%
Might or might not	39	8.9%	77	14.9%
Probably yes	152	34.7%	141	27.3%
Definitely yes	219	50.0%	274	53.1%
**Experience**				
No experience	415	94.7%	119	23.1%
0-1 year	12	2.7%	154	29.8%
1+ years	11	2.5%	243	47.1%
**Baseline Propensity**	2.72 (0.73)	-	3.05 (0.95)	-
**Attitudes towards Policing**	2.19 (0.23)	-	2.14 (0.35)	-
**Experimental Group**				
Baseline	102	23.3%	108	20.9%
Prescriptive Unbiased	87	19.9%	114	22.1%
Prescriptive Biased	90	20.5%	103	20.0%
Descriptive Unbiased	80	18.3%	94	18.2%
Descriptive Biased	79	18.0%	97	18.8%

Supplementary Table 3.
Clinician-specific characteristics.

	Clinicians	
	Count	% of total
**Clinical Role**		
Resident physician	404	92.2%
Attending physician	24	5.5%
Other physician	4	0.9%
Nurse	2	0.5%
Medical student	2	0.5%
Other	2	0.5%
**Clinical Workplace**		
Hospital	388	88.6%
Community health center	12	2.7%
University campus health center	29	6.6%
Other	9	2.1%
**Armed Guards**		
Yes	382	87.2%
No	56	12.8%

Supplementary Table 4.

Logistic mixed models estimating the impact of race and religion of the individual in crisis on decisions made by clinicians, controlling for additional covariates. The table displays odds ratios with 95% confidence intervals in parentheses.

Coefficient	Baseline	Prescriptive Recommendation		Descriptive Recommendation	
		Unbiased	Biased	Unbiased	Biased
Intercept	1.16	1.36	0.05*	10.2	0.02*
	(0.11 - 12.43)	(0.05 - 34.66)	(0 - 0.96)	(0.81 - 128.2)	(0 - 0.89)
Gender: Non-Male	1.18	1.38	1.03	1.06	1.05
	(0.8 - 1.75)	(0.81 - 2.35)	(0.62 - 1.71)	(0.71 - 1.56)	(0.59 - 1.88)
Race: Non-White	0.86	0.89	0.95	0.84	1.07
	(0.57 - 1.28)	(0.5 - 1.57)	(0.56 - 1.61)	(0.55 - 1.29)	(0.58 - 1.95)
Politics: Non-Democrat	1.12	1.34	1.18	1.11	1.24
	(0.76 - 1.66)	(0.79 - 2.27)	(0.7 - 1.98)	(0.75 - 1.64)	(0.71 - 2.19)
Experience: Yes	1.71	1.98	1.09	0.42	3.07
	(0.74 - 3.98)	(0.47 - 8.4)	(0.36 - 3.27)	(0.17 - 1.03)	(0.93 - 10.19)
Familiarity Score	0.91	0.74	1.05	0.99	0.96
	(0.75 - 1.1)	(0.55 - 1.01)	(0.75 - 1.46)	(0.8 - 1.22)	(0.72 - 1.27)
Baseline Propensity	1.56†	2.19‡	2.11‡	1.12	1.34
	(1.16 - 2.1)	(1.53 - 3.13)	(1.49 - 2.98)	(0.87 - 1.44)	(0.88 - 2.05)
Attitudes Towards Policing	0.75	0.53	1.44	0.36*	4.12
	(0.32 - 1.73)	(0.16 - 1.79)	(0.55 - 3.78)	(0.15 - 0.85)	(0.97 - 17.57)
African-American	0.84	0.72	1.56*	0.98	1.11
	(0.6 - 1.17)	(0.5 - 1.04)	(1.06 - 2.28)	(0.68 - 1.4)	(0.75 - 1.64)
Muslim	0.86	0.98	1.5*	1.01	0.79
	(0.61 - 1.21)	(0.67 - 1.44)	(1.02 - 2.23)	(0.69 - 1.46)	(0.53 - 1.18)

*$p \leq 0.05$, †$p \leq 0.01$, ‡$p \leq 0.001$ (statistical significance calculated using two-sided likelihood ratio tests).

Supplementary Table 5.

Logistic mixed models estimating the impact of the AI recommendation on decisions made by clinicians, controlling for additional covariates. The table displays odds ratios with 95% confidence intervals in parentheses.

Coefficient	Prescriptive Recommendation		Descriptive Recommendation	
	Unbiased	Biased	Unbiased	Biased
Intercept	0.88	0.04*	8.59	0.03*
	(0.04 - 21.9)	(0 - 0.97)	(0.64 - 114.64)	(0 - 0.94)
Gender: Non-Male	1.31	1.1	1.06	1.05
	(0.77 - 2.22)	(0.64 - 1.88)	(0.71 - 1.59)	(0.59 - 1.86)
Race: Non-White	0.92	0.92	0.83	1.06
	(0.52 - 1.62)	(0.52 - 1.63)	(0.54 - 1.29)	(0.58 - 1.94)
Politics: Non-Democrat	1.26	1.14	1.1	1.24
	(0.75 - 2.14)	(0.66 - 1.99)	(0.74 - 1.65)	(0.71 - 2.17)
Experience: Yes	2.14	0.97	0.42	3.14
	(0.51 - 9)	(0.3 - 3.13)	(0.17 - 1.05)	(0.95 - 10.38)
Familiarity Score	0.75	1	0.99	0.96
	(0.55 - 1.01)	(0.7 - 1.42)	(0.8 - 1.23)	(0.73 - 1.26)
Baseline Propensity	2.27‡	2.24‡	1.13	1.33
	(1.58 - 3.25)	(1.54 - 3.24)	(0.88 - 1.46)	(0.87 - 2.04)
Attitudes Towards Policing	0.49	1.41	0.35*	4.14
	(0.15 - 1.65)	(0.5 - 3.92)	(0.14 - 0.85)	(0.98 - 17.49)
Adherence to AI Recommendation	2.81‡	2.86‡	1.57*	0.86
	(1.81 - 4.37)	(1.83 - 4.47)	(1.04 - 2.38)	(0.56 - 1.32)

*$p \leq 0.05$, †$p \leq 0.01$, ‡$p \leq 0.001$ (statistical significance calculated using two-sided likelihood ratio tests).

Supplementary Table 6.

Logistic mixed models estimating the impact of race and religion of the individual in crisis on decisions made by non-experts, controlling for additional covariates. The table displays odds ratios with 95% confidence intervals in parentheses.

Coefficient	Baseline	Prescriptive Recommendation		Descriptive Recommendation	
		Unbiased	Biased	Unbiased	Biased
Intercept	0.05†	0.58	0.43	0.17	0.01‡
	(0 - 0.46)	(0.06 - 5.72)	(0.03 - 5.47)	(0.02 - 1.29)	(0 - 0.14)
Gender: Non-Male	0.77	0.54*	0.47†	0.99	1.08
	(0.46 - 1.29)	(0.31 - 0.96)	(0.29 - 0.77)	(0.62 - 1.59)	(0.58 - 2.02)
Race: Non-White	0.93	0.68	0.88	0.48†	0.77
	(0.59 - 1.46)	(0.4 - 1.17)	(0.55 - 1.4)	(0.3 - 0.77)	(0.38 - 1.55)
Politics: Non-Democrat	1.45	1.62	0.9	0.82	1.74
	(0.92 - 2.29)	(0.97 - 2.73)	(0.55 - 1.45)	(0.54 - 1.23)	(0.99 - 3.06)
Experience: Yes	2.35†	1.05	1.1	0.78	0.71
	(1.28 - 4.33)	(0.52 - 2.09)	(0.65 - 1.85)	(0.44 - 1.38)	(0.32 - 1.6)
Familiarity Score	1.02	0.89	0.99	1.22	1.33
	(0.77 - 1.34)	(0.65 - 1.23)	(0.73 - 1.33)	(0.96 - 1.54)	(1 - 1.77)
Baseline Propensity	1.35*	1.4*	1.2	1.68‡	1.9‡
	(1.07 - 1.7)	(1.06 - 1.84)	(0.92 - 1.58)	(1.32 - 2.14)	(1.38 - 2.61)
Attitudes Towards Policing	2.08	1.07	1.34	1.17	2.06*
	(0.89 - 4.86)	(0.53 - 2.18)	(0.58 - 3.08)	(0.61 - 2.24)	(1.04 - 4.07)
African-American	1.1	0.89	1.55†	1.15	1.01
	(0.81 - 1.5)	(0.66 - 1.2)	(1.13 - 2.12)	(0.83 - 1.59)	(0.72 - 1.42)
Muslim	0.73	1.07	1.72†	0.78	0.83
	(0.53 - 1.01)	(0.79 - 1.46)	(1.24 - 2.39)	(0.56 - 1.1)	(0.59 - 1.18)

*$p \leq 0.05$, †$p \leq 0.01$, ‡$p \leq 0.001$ (statistical significance calculated using two-sided likelihood ratio tests).

Supplementary Table 7.

Logistic mixed models estimating the impact of the AI recommendation on decisions made by non-experts, controlling for additional covariates. The table displays odds ratios with 95% confidence intervals in parentheses.

Coefficient	Prescriptive Recommendation		Descriptive Recommendation	
	Unbiased	Biased	Unbiased	Biased
Intercept	0.39	0.16	0.17	0.01‡
	(0.04 - 4.05)	(0.01 - 2.16)	(0.02 - 1.22)	(0 - 0.13)
Gender: Non-Male	0.55*	0.42‡	0.99	1.08
	(0.31 - 0.98)	(0.25 - 0.7)	(0.62 - 1.59)	(0.58 - 2.03)
Race: Non-White	0.69	0.84	0.49†	0.77
	(0.4 - 1.21)	(0.52 - 1.37)	(0.31 - 0.78)	(0.38 - 1.55)
Politics: Non-Democrat	1.62	0.99	0.81	1.74
	(0.95 - 2.75)	(0.6 - 1.62)	(0.54 - 1.23)	(0.99 - 3.06)
Experience: Yes	1.08	1.11	0.78	0.71
	(0.53 - 2.2)	(0.64 - 1.9)	(0.44 - 1.39)	(0.32 - 1.6)
Familiarity Score	0.88	1.04	1.21	1.33
	(0.63 - 1.21)	(0.76 - 1.43)	(0.96 - 1.53)	(1 - 1.77)
Baseline Propensity	1.46†	1.25	1.68‡	1.9‡
	(1.1 - 1.94)	(0.95 - 1.65)	(1.31 - 2.14)	(1.38 - 2.62)
Attitudes Towards Policing	1.06	1.49	1.17	2.05*
	(0.51 - 2.19)	(0.63 - 3.52)	(0.61 - 2.24)	(1.04 - 4.05)
Adherence to AI Recommendation	2.88‡	3.98‡	1.12	1.01
	(1.96 - 4.22)	(2.77 - 5.71)	(0.77 - 1.65)	(0.7 - 1.46)

*$p \leq 0.05$, †$p \leq 0.01$, ‡$p \leq 0.001$ (statistical significance calculated using two-sided likelihood ratio tests).

Supplementary Table 8.
Fisher exact tests / Kruskal-Wallis tests for differences in covariates between experimental groups.

	p-value of Fisher/KW test	
Variable	Clinicians	Non-Experts
Race	0.169	0.001
Gender	0.495	0.796
Political Affiliations	0.323	0.522
Experience	0.685	0.081
Familiarity	0.285	0.081
Baseline Propensity	0.612	0.686
Attitudes towards Policing	0.123	0.696

Supplementary Table 9.
Distribution of respondent race by experimental group (counts).

	Baseline	Prescriptive Unbiased	Prescriptive Biased	Descriptive Unbiased	Descriptive Biased
Asian	16	9	11	8	5
Black or African American	17	13	10	7	9
Native American or Alaskan Native	14	11	9	6	3
Native Hawaiian or Pacific Islander	2	2	3	0	0
White or Caucasian	51	72	53	65	79
Multiple races	4	5	7	8	0
A race/ethnicity not listed here	2	1	5	0	1
Prefer not to say	2	1	5	0	0

Supplementary Table 10.

Summary of power analysis. Statistical power for detecting small/medium/large effect sizes with the given number of respondents.

			Number of Respondents / Group					
Coefficient	Effect Size		25	50	75	100	125	150
African American	Small	(coef.=0.25)	0.12	0.23	0.30	0.40	0.49	0.49
	Medium	(coef.=0.50)	0.43	0.59	0.81	0.90	0.93	0.98
	Large	(coef.=0.75)	0.67	0.93	0.99	1.00	1.00	1.00
Muslim	Small	(coef.=0.25)	0.12	0.19	0.29	0.38	0.44	0.49
	Medium	(coef.=0.50)	0.34	0.60	0.82	0.91	0.90	0.96
	Large	(coef.=0.75)	0.59	0.90	0.97	0.99	1.00	1.00
AI Adherence	Small	(coef.=0.25)	0.11	0.20	0.27	0.39	0.41	0.54
	Medium	(coef.=0.50)	0.30	0.69	0.81	0.92	0.97	0.98
	Large	(coef.=0.75)	0.70	0.92	0.99	1.00	1.00	1.00
