# Supplementary material for: Mitigating the impact of biased artificial intelligence in emergency decision-making
Source: Commun Med (Lond). 2022 Nov 21;2:149. doi: 10.1038/s43856-022-00214-4 (PMC9681767; doi:10.1038/s43856-022-00214-4)
Supplement: Supplementary file 3 — Reporting Summary [file 43856_2022_214_MOESM3_ESM.pdf]

nature research

Corresponding author(s): Hammaad Adam

Last updated by author(s): Oct 27, 2022

Reporting Summary

Nature Research wishes to improve the reproducibility of the work that we publish. This form provides structure for consistency and transparency in reporting. For further information on Nature Research policies, see our Editorial Policies and the Editorial Policy Checklist.

Statistics

For all statistical analyses, confirm that the following items are present in the figure legend, table legend, main text, or Methods section.

n/a	Confirmed	
☐	☒	The exact sample size (n) for each experimental group/condition, given as a discrete number and unit of measurement
☒	☐	A statement on whether measurements were taken from distinct samples or whether the same sample was measured repeatedly
☐	☒	The statistical test(s) used AND whether they are one- or two-sided<br>*Only common tests should be described solely by name; describe more complex techniques in the Methods section.*
☐	☒	A description of all covariates tested
☒	☐	A description of any assumptions or corrections, such as tests of normality and adjustment for multiple comparisons
☐	☒	A full description of the statistical parameters including central tendency (e.g. means) or other basic estimates (e.g. regression coefficient) AND variation (e.g. standard deviation) or associated estimates of uncertainty (e.g. confidence intervals)
☐	☒	For null hypothesis testing, the test statistic (e.g. F, t, r) with confidence intervals, effect sizes, degrees of freedom and P value noted<br>*Give P values as exact values whenever suitable.*
☒	☐	For Bayesian analysis, information on the choice of priors and Markov chain Monte Carlo settings
☐	☒	For hierarchical and complex designs, identification of the appropriate level for tests and full reporting of outcomes
☒	☐	Estimates of effect sizes (e.g. Cohen's d, Pearson's r), indicating how they were calculated

Our web collection on statistics for biologists contains articles on many of the points above.

Software and code

Policy information about availability of computer code

Data collection	Qualtrics
Data analysis	The free programming languages R (3.6.3) was used to perform all statistical analyses. Code to reproduce the paper’s main findings can be found at https://doi.org/10.5281/zenodo.7293263

For manuscripts utilizing custom algorithms or software that are central to the research but not yet described in published literature, software must be made available to editors and reviewers. We strongly encourage code deposition in a community repository (e.g. GitHub). See the Nature Research guidelines for submitting code & software for further information.

Data

Policy information about availability of data

All manuscripts must include a data availability statement. This statement should provide the following information, where applicable:

- Accession codes, unique identifiers, or web links for publicly available datasets
- A list of figures that have associated raw data
- A description of any restrictions on data availability

Anonymized versions of the datasets collected and analyzed during the current study are publicly available at https://doi.org/10.5281/zenodo.7293263

Field-specific reporting

Please select the one below that is the best fit for your research. If you are not sure, read the appropriate sections before making your selection.

☐ Life sciences ☒ Behavioural & social sciences ☐ Ecological, evolutionary & environmental sciences

For a reference copy of the document with all sections, see nature.com/documents/nr-reporting-summary-flat.pdf

Behavioural & social sciences study design

All studies must disclose on these points even when the disclosure is negative.

Study description	Quantitative experiment conducted online to assess the impact of biased AI on emergency decision making
Research sample	Our study had two samples: clinicians and non-experts. The type of decision our study considers may need to be performed by any individual, regardless of occupation. However, we hypothesized that clinical expertise may influence decision making in mental health emergencies. Thus, we tested our hypotheses in both these groups of participants.
Sampling strategy	We used a convenience sample for our analysis. We determined sample size using a simulation-based power analysis (see Supplementary Methods).
Data collection	Our experiment was web-based and conducted using Qualtrics. Clinicians were recruited by emailing staff and residents at hospitals in the United States and Canada, while non-experts were recruited through social media (Facebook, Reddit) and university email lists.
Timing	May 2021 - December 2021
Data exclusions	We excluded participants who Qualtrics identified as bots (using the QRecaptcha score), as well as those who rushed through our survey (finishing in <5 minutes). We also excluded duplicate responses from the same participant. This excluded 15 clinician and 2,347 non-expert responses.
Non-participation	Two non-experts and no clinicians explicitly declined to participate.
Randomization	Participants were randomly assigned into experimental groups using Qualtrics' randomization functionality.

Reporting for specific materials, systems and methods

We require information from authors about some types of materials, experimental systems and methods used in many studies. Here, indicate whether each material, system or method listed is relevant to your study. If you are not sure if a list item applies to your research, read the appropriate section before selecting a response.

Materials & experimental systems

n/a	Involved in the study	
☒	☐	Antibodies
☒	☐	Eukaryotic cell lines
☒	☐	Palaeontology and archaeology
☒	☐	Animals and other organisms
☐	☒	Human research participants
☒	☐	Clinical data
☒	☐	Dual use research of concern

Methods

n/a	Involved in the study	
☒	☐	ChIP-seq
☒	☐	Flow cytometry
☒	☐	MRI-based neuroimaging

Human research participants

Policy information about studies involving human research participants

Population characteristics	See Supplementary Table 1 for a detailed summary.
Recruitment	Clinicians were recruited by emailing staff and residents at hospitals in the United States and Canada, while non-experts were recruited through social media (Facebook, Reddit) and university email lists.
Ethics oversight	MIT Committee on the Use of Humans as Experimental Subjects (COUHES). This study was determined to be exempt from a full ethical review, because it met the criteria for exemption defined in Federal regulation 45 CFR 46.

Note that full information on the approval of the study protocol must also be provided in the manuscript.
