# Supplementary material for: Mitigating the impact of biased artificial intelligence in emergency decision-making
Source: Commun Med (Lond). 2022 Nov 21;2:149. doi: 10.1038/s43856-022-00214-4 (PMC9681767; doi:10.1038/s43856-022-00214-4)
Supplement: Supplementary file 2 — Peer Review File [file 43856_2022_214_MOESM2_ESM.pdf]

nature portfolio

Peer Review File

Mitigating the impact of biased artificial intelligence in emergency decision-making

Open Access This file is licensed under a Creative Commons Attribution 4.0 International License, which permits use, sharing, adaptation, distribution and reproduction in any medium or format, as long as you give appropriate credit to the original author(s) and the source, provide a link to the Creative Commons license, and indicate if changes were made. In the cases where the authors are anonymous, such as is the case for the reports of anonymous peer reviewers, author attribution should be to 'Anonymous Referee' followed by a clear attribution to the source work. The images or other third party material in this file are included in the article's Creative Commons license, unless indicated otherwise in a credit line to the material. If material is not included in the article's Creative Commons license and your intended use is not permitted by statutory regulation or exceeds the permitted use, you will need to obtain permission directly from the copyright holder. To view a copy of this license, visit http://creativecommons.org/licenses/by/4.0/.

Reviewers' comments:

Reviewer #1 (Remarks to the Author):

The study investigates the problem of automation bias with a view on fairness/discrimination issues. To this end, the investigators conducted an empirical study in which a biased algorithm induced unfair decisions, impacting African-American and Muslim individuals. It was also found that the effects can be mitigated by switching from a prescriptive recommendations to a descriptive language.

The study is overall interesting, even though the results are not particularly surprising, given the previous studies by Tschandl et al (which should be cited btw) and Gaube et al. The formal study design and the statistical analysis strike me as solid.

My concern is the following: What can we learn from the study for more realistic clinical settings? There are two aspects in the information presentation by the algorithm that are likely to reinforce biases in the human decision-maker. First, the race/religion has been made salient early on. In ordinary emergency hotline scenarios, this shoud rarely ever be the case. Rather than emphasizing the race/religion, it would have been interesting if the investigators had used proxies -- e.g. certain surnames that are associated with the membership in a marginalized social group.

Second, while the differences in effect between prescriptive and descriptive language are interesting, I wonder whether it is realistic that we will ever use risk-prediction models that make prescriptive recommendations. Furthermore, it is of course clear that a prescriptive language increases the likelihood that human decision-makers adhere to algorithmic recommendations.

In sum, I think that certain methodological choices need a thorough justification.

Reviewer #2 (Remarks to the Author):

Adam et al. present an intriguing and important experiment showing how end users can blindly follow the suggestion of AI. An important implication of this, is when the AI presents demographically biased recommendation, unbiased users will conduct biased actions. This is an important contribution to the ML and Clinical Decision Making fields.

I have some suggestions both analytically and framing.

Analytically:
* I would suggest not using logistic regression models, but instead use log-regression or log-linear models with robust variance estimates. In this setting the OR is a hard parameter to interpret and would be better if the effects could be interpreted as RR or AR. Additionally, I would not report p-values in the manuscript. Instead please report effect estimates with confidence intervals.

* In the mixed effects model I'm confused by the individual level propensity variable. Wouldn't that be accounted for and hence colinear with a random effect term?

Framing:

* While bias is obviously a very important topic I'm wondering if this experiment has broader implications that are being ignored. Namely, the blind following of AI models. The results seem to suggest that end users follow the recommendations of AI. This can be problematic from a racial bias perspective but is also problematic more broadly since all models are wrong to some degree even if they are not racially biased.

* Related to the above, it would be worth tying the "style" findings in with recent recommendations by the FDA about the necessity for model interpretability. The results seem to suggest that an interpretable model that was clear on why recommendations were generated would be able to mitigate such biases. I'd be curious on the authors thoughts on this.

Reviewer #3 (Remarks to the Author):

In this paper the authors claim that AI results can significantly bias individual decisions.

The manuscript needs to explain better what biased vs. unbiased AI means and how it is actually implemented.

Pg 5. From the text and figure, descriptive recommendations are not very clear. Is the flag only provided when there is risk for violence, and there is no flag otherwise?

It would be good to have a table with prescriptive and descriptive odds ratio results. Currently comprehensive OR results are missing from the descriptive case.

Minor:
1) Intro pg 4, “yielded” is not used correctly, should be removed.
2) Discussion reference 18 is missing brackets.

Response Letter
Just Following AI Orders: When Unbiased People Are Influenced By Biased AI

We would like to thank the editorial team and the reviewers for their detailed consideration of our work. We found the reviewers' comments very helpful, and have significantly revised the paper in line with their suggestions.

We summarize the main changes in our point-by-point response to the reviewers below (including the reviewers' comments *in italics* for reference). In the revised paper, we have highlighted all changes in yellow.

Response to Reviewer #1

R1.1: "The study investigates the problem of automation bias with a view on fairness/discrimination issues. To this end, the investigators conducted an empirical study in which a biased algorithm induced unfair decisions, impacting African-American and Muslim individuals. It was also found that the effects can be mitigated by switching from a prescriptive recommendations to a descriptive language.

The study is overall interesting, even though the results are not particularly surprising, given the previous studies by Tschandl et al (which should be cited btw) and Gaube et al. The formal study design and the statistical analysis strike me as solid."

Thank you for your encouragement and interest in our work. We appreciate your detailed feedback; we have revised the paper in line with your recommendations, and believe these changes have made the paper stronger. We have cited the Tschandl et al paper in the revised version. We address your comments in further detail below.

R1.2: "My concern is the following: What can we learn from the study for more realistic clinical settings? There are two aspects in the information presentation by the algorithm that are likely to reinforce biases in the human decision-maker. First, the race/religion has been made salient early on. In ordinary emergency hotline scenarios, this should rarely ever be the case. Rather than emphasizing the race/religion, it would have been interesting if the investigators had used proxies -- e.g. certain surnames that are associated with the membership in a marginalized social group."

We agree that indicating race and religion in each vignette explicitly in text form may not happen in a realistic hotline scenario. It is also possible that emphasizing race and religion in this way could reinforce biases in some settings. However, despite our explicit communication of race and religion in this study, we did not observe that participant responses were biased without an AI recommendation, as would be expected if their biases were being reinforced. Rather, we find that respondents were biased only when they were presented with prescriptive recommendations from a biased AI model. Importantly, this means that communicating race and religion to participants is less important than models making biased recommendations with access to these sensitive attributes. Recent work has shown that AI models can infer sensitive attributes from other medical data with high performance (1, 2). Our main finding - that people (both experts and non-experts) follow biased AI advice when it is given in a prescriptive way - must be

carefully considered in the many real-world clinical scenarios where biased models may be used to inform important decisions.

Using a subtler instrument like names to elicit a respondent's implicit biases–which are more likely to drive behavior under time pressure, ambiguity, and cognitive load (3)–is an important avenue for future work. We did not investigate this in our work, and agree that it may lead to more disparate decision making. We have recognized this as a limitation in the revised version.

1. Gichoya, Judy Wawira, et al. "AI recognition of patient race in medical imaging: a modelling study." *The Lancet Digital Health* (2022).
2. Adam, Hammaad, et al. "Write It Like You See It: Detectable Differences in Clinical Notes By Race Lead To Differential Model Recommendations." *AAAI/ACM Conference on AI, Ethics, and Society* (2022).
3. Bertrand, Marianne, and Esther Duflo. "Field experiments on discrimination." *Handbook of economic field experiments* 1 (2017): 309-393.

R1.3: "Second, while the differences in effect between prescriptive and descriptive language are interesting, I wonder whether it is realistic that we will ever use risk-prediction models that make prescriptive recommendations. Furthermore, it is of course clear that a prescriptive language increases the likelihood that human decision-makers adhere to algorithmic recommendations.

In sum, I think that certain methodological choices need a thorough justification."

The use of AI to support high-stakes decision making is still in its early stages, and there is no standard way to display model recommendations. The few examples of such systems in practice (4, 5, 6) usually communicate model recommendations through the form of risk scores. Risk scores are similar to our descriptive flags in that they indicate the underlying risk of some event, but do not make an explicit recommendation. However, risk scores have been mapped to specific actions in some model deployment settings, such as pretrial release decisions in criminal justice where risk scores (e.g. COMPAS (5)) are mapped to actionable recommendations (4). Even more directly, many machine learning models directly predict a clinical intervention or action (e.g., intubation, fluid administration, etc.) (7,8) or a triage condition (e.g. more screening is not needed for healthy chest x-rays) (9). The FDA has also recently approved models that automatically make diagnostic recommendations to clinical staff (10). These settings are similar to our prescriptive setting, as recommendations correspond to a specific action. In our experiment, we displayed prescriptive recommendations and descriptive flags (i.e. instead of risk scores with or without a suggested action map), to minimize respondent fatigue and to ensure that our advice was accessible for both clinicians and non-experts.

We appreciate that the lessons from our experiment should be considered in the context of more realistic clinical settings. We believe that our main finding is very practically applicable to designers, deployers, users and auditors of models - prescriptive recommendations greatly increased algorithmic adherence, while descriptive flags did not have the same effect. The key points made in our discussion section—on the ease of biasing AI models, the need to test human-AI factors like recommendation style, and the dangers of using models without extensive internal and external validation—also generalize to real concerns about deployments of AI systems in medicine (11, 12).

We have included these parallels in the revised paper.

4. Chohlas-Wood, Alex. "Understanding risk assessment instruments in criminal justice." *Brookings Institution's series on AI and Bias* (2020).
5. Dieterich, William, Christina Mendoza, and Tim Brennan. "COMPAS risk scales: Demonstrating accuracy equity and predictive parity." *Northpointe Inc* 7.4 (2016).
6. Tschandl, Philipp, et al. "Human–computer collaboration for skin cancer recognition." *Nature Medicine* 26.8 (2020): 1229-1234.
7. Topol, Eric J. "High-performance medicine: the convergence of human and artificial intelligence." *Nature medicine* 25.1 (2019): 44-56.
8. Ghassemi, Marzyeh, et al. "Predicting intervention onset in the ICU with switching state space models." *AMIA Summits on Translational Science Proceedings* 2017 (2017): 82.
9. Seyyed-Kalantari, Laleh, et al. "Underdiagnosis bias of artificial intelligence algorithms applied to chest radiographs in under-served patient populations." *Nature medicine* 27.12 (2021): 2176-2182.
10. Abràmoff, Michael D., et al. "Pivotal trial of an autonomous AI-based diagnostic system for detection of diabetic retinopathy in primary care offices." *NPJ digital medicine* 1.1 (2018): 1-8.
11. Keane, Pearse A., and Eric J. Topol. "With an eye to AI and autonomous diagnosis." *NPJ Digital Medicine* 1.1 (2018): 1-3.
12. Wiens, Jenna, et al. "Do no harm: a roadmap for responsible machine learning for health care." *Nature medicine* 25.9 (2019): 1337-1340.

<u>Response to Reviewer #2</u>

R2.1: "Adam et al. present an intriguing and important experiment showing how end users can blindly follow the suggestion of AI. An important implication of this, is when the AI presents demographically biased recommendation, unbiased users will conduct biased actions. This is an important contribution to the ML and Clinical Decision Making fields.

I have some suggestions both analytically and framing."

Thank you for your encouragement and support for our work. We greatly appreciate your feedback on our analytical approach and framing. We have revised the paper in line with your suggestions, and believe these changes have made the paper stronger. We address each of your comments below in further detail.

R2.2: "Analytically:
** I would suggest not using logistic regression models, but instead use log-regression or log-linear models with robust variance estimates. In this setting the OR is a hard parameter to interpret and would be better if the effects could be interpreted as RR or AR. Additionally, I would not report p-values in the manuscript. Instead please report effect estimates with confidence intervals."*

Your point on relative risk vs. odds ratio is well appreciated. We describe below in detail our reasoning for using logistic regression in our analysis. We followed Reviewer #3's suggestions to replace regression coefficients with odds ratios in the main table, and have clarified in the

manuscript that the results display odds ratios, not relative risk. We have also replaced p-values with 95% confidence intervals wherever possible.

Logistic regression is best suited to our modeling needs because our outcome is binary (0 or 1) and is not rare. Unfortunately, both log binomial and log Poisson modeling approaches are not well-suited to our experiment. First, log binomial models often fail to converge when using real data (13, 14) even when more stable fitting procedures are used (15). Second, log Poisson regression with robust standard errors yield conservative standard errors, as the model is misspecified for binary outcomes (14). Prior work has also highlighted the conservatism of modified log Poisson regression —particularly for small samples—due to the properties of the sandwich estimator for standard errors (16).

For completeness, we attempted to model the relative risk directly using both log binomial regression and log Poisson regression with robust standard errors. Our log binomial models did not converge when using the cited stable fitting procedures. While using robust instead of non-robust standard errors with log Poisson regression reduces the impact of the cited misspecification, we still found such inference to be too conservative for our sample size. To demonstrate the modified Poisson regression's conservatism, we re-ran our power analysis using both methods—modified Poisson and logistic regression—for medium sized effects. Here, medium effect refers to a linear coefficient of 0.5 (odds ratio of 1.65); the observed effects in our study were in this range, though slightly smaller. We simulated data using a similar structure as in our experiment, and calculated power over 1,000 replications. Modified Poisson regression displayed significantly lower power for medium-sized effects than the logistic regression (Table A).

Table A.
Statistical power for detecting medium effect sizes with the given number of respondents for logistic regression and the modified Poisson procedure. Power is defined as the fraction of replications in which the model identifies a statistically significant effect (significance level = 0.05, 1,000 total replications).

		Number of Respondents / Group	
Coefficient	Method	75	100
African American	Modified Poisson Mixed Effects	0.65	0.78
	Logistic Mixed Effects	0.81	0.91
Muslim	Modified Poisson Mixed Effects	0.65	0.76
	Logistic Mixed Effects	0.79	0.88
AI Adherence	Modified Poisson Mixed Effects	0.69	0.82
	Logistic Mixed Effects	0.80	0.90

13. Williamson, Tyler, Misha Eliasziw, and Gordon Hilton Fick. "Log-binomial models: exploring failed convergence." *Emerging themes in epidemiology* 10.1 (2013): 1-10.

14. Zou, Guangyong. "A modified poisson regression approach to prospective studies with binary data." *American journal of epidemiology* 159.7 (2004): 702-706.
15. Donoghoe, Mark W., and Ian C. Marschner. "logbin: An R package for relative risk regression using the log-binomial model." *Journal of Statistical Software* 86 (2018): 1-22.
16. Chen, Wansu, et al. "Comparing performance between log-binomial and robust Poisson regression models for estimating risk ratios under model misspecification." *BMC medical research methodology* 18.1 (2018): 1-12.

R2.3: " In the mixed effects model I'm confused by the individual level propensity variable. Wouldn't that be accounted for and hence colinear with a random effect term?"*

The respondent's baseline propensity to call the police is measured before the main experiment in a separate set of questions (further detail in the Supplementary Text). Each respondent's propensity was summarized on a scale of 1 to 6; this variable was then included as a covariate. As constructed, this approach should not be co-linear with the random intercept term. However, we agree that the purpose of including this term is served by the random intercept. In the revised set of results, we have removed the baseline propensity term: the models now control only for the respondent and vignette-level random effects. This change made a negligible impact on the coefficients of interest (Tables 1a and 1b in the revised paper). We still include baseline propensity as a covariate in the appendix results that control for additional respondent characteristics (Tables S4-S7 in the supplement). These results are also negligibly changed.

R2.4: "Framing:
** While bias is obviously a very important topic I'm wondering if this experiment has broader implications that are being ignored. Namely, the blind following of AI models. The results seem to suggest that end users follow the recommendations of AI. This can be problematic from a racial bias perspective but is also problematic more broadly since all models are wrong to some degree even if they are not racially biased.*

We were hesitant to make strong claims about advice style interacting with model accuracy as in our experiment, there is no ground truth. While it is clearly wrong to always advise participants to call for police based on demographics, the decisions made by our participants (i.e. to call for police or medical help) are subjective, and we only identify bias by comparing the rates of calling the police for different participant identities (Caucasian vs. African-American, Muslim vs. non-Muslim).

We agree that one of our most interesting findings is that decision makers may adhere strongly to inaccurate models if recommendations are displayed prescriptively, and have now included this in our discussion section. We thank you for this suggestion - it was very thought-provoking and has re-framed our thinking on future work extending our findings.

*R2.5 " * Related to the above, it would be worth tying the "style" findings in with recent recommendations by the FDA about the necessity for model interpretability. The results seem to suggest that an interpretable model that was clear on why recommendations were generated would be able to mitigate such biases. I'd be curious on the authors thoughts on this.*

Model interpretability is indeed an important topic area in machine learning and health. We believe that interpretability alone is insufficient to make AI deployments robust against inaccuracy and bias. Recent work has noted that explanation methods in health care settings may be poorly suited to domain needs (17), and common explanation methods themselves can increase model bias, i.e. the gap in performance between the worst and best subgroups (18). Other work, outside of healthcare, has found that increasing model interpretability reduces human ability to identify model mistakes due to information overload (19).

In line with your recommendation, we have tied our findings on style to the FDA guidance for good machine learning development. We position our recommendation within the FDA's emphasis on "human considerations" when designing AI systems to inform human decisions, and emphasize that such human considerations—including recommendation style and interpretability—must undergo thorough validation and testing before deployment.

We have edited the text to emphasize these points in our conclusion.

17. Ghassemi, Marzyeh, Luke Oakden-Rayner, and Andrew L. Beam. "The false hope of current approaches to explainable artificial intelligence in healthcare." *The Lancet Digital Health* 3.11: e745-e750. 2021
18. Balagopalan, Aparna, et al. "The Road to Explainability is Paved with Bias: Measuring the Fairness of Explanations." *In 2022 ACM Conference on Fairness, Accountability, and Transparency (FAccT '22).* 2022.
19. Poursabzi-Sangdeh, Forough, et al. "Manipulating and measuring model interpretability." *Proceedings of the 2021 CHI conference on human factors in computing systems*. 2021.

<u>Response to Reviewer #3</u>

R3.1: "In this paper the authors claim that AI results can significantly bias individual decisions.

The manuscript needs to explain better what biased vs. unbiased AI means and how it is actually implemented."

Thank you for your feedback. We appreciate your interest in our work, and have revised the paper in line with your recommendations.

We have added language to the paper that clarifies the meaning of a biased vs. unbiased model. In short, a biased model is more likely to recommend police help (vs. medical help) if the described individual is African-American or Muslim, while the unbiased model is equally likely to suggest police help for both race and religion groups. Fig S1 in the supplement describes the process of model biasing in further depth: we have added a summary of this procedure and a reference to the figure in the main text.

R3.2: "Pg 5. From the text and figure, descriptive recommendations are not very clear. Is the flag only provided when there is risk for violence, and there is no flag otherwise?"

Your interpretation is correct: we display a flag if the model predicts risk of violence, and do not display a flag otherwise. We have updated the figure and text to make this more clear.

R3.3: "It would be good to have a table with prescriptive and descriptive odds ratio results. Currently comprehensive OR results are missing from the descriptive case."

In line with your recommendation, we now use the tables in the main text to display odds ratios (with 95% CI's) instead of regression coefficients. We hope that this facilitates an easier interpretation of our results.

R3.4: "Minor:
1) Intro pg 4, "yielded" is not used correctly, should be removed.
2) Discussion reference 18 is missing brackets."

Thank you for pointing out these issues. They have been corrected in the manuscript.

REVIEWERS' COMMENTS:

Reviewer #1 (Remarks to the Author):

Dear author,

While I was pretty skeptical of the first version of the paper, I like the revised version. This is particularly due to the fact that the paper is very thouughtful about its methodological constraints and the inferences that can be drawn from the study. In my view, the paper is now good enough to be published.

Reviewer #2 (Remarks to the Author):

The authors did a very nice job responding to comments.

Reviewer #3 (Remarks to the Author):

The authors have thoughtfully revised the manuscript and provided new information that help to clarify the work. Couple of further recommendations are as below -
In the new tables, it is not clear what the likelihood ratio test is comparing. The likelihood-ratio test assesses the goodness of fit of two competing statistical models based on the ratio of their likelihoods, so is each model significance relative to the baseline? Please clarify.

For Table 1b it would be good to reiterate that the OR is different than in Table 1a, i.e. in this case it is the OR with regards to adhering to the model's decision rather than the OR to call the police. Else it can be confusing. The observation that clinicians adhere significantly to the descriptive unbiased model choice but not to the biased model choice is puzzling. What explanation can the authors provide for the descriptive biased model not showing significance for clinicians?

Response Letter

We would like to thank the editorial team and the reviewers for their support of our work. We summarize the main changes in our point-by-point response to the reviewers below (including the reviewers' comments *in italics* for reference).

Response to Reviewer #1

Dear author,

While I was pretty skeptical of the first version of the paper, I like the revised version. This is particularly due to the fact that the paper is very thoughtful about its methodological constraints and the inferences that can be drawn from the study. In my view, the paper is now good enough to be published.

Thank you for your encouragement. We greatly appreciated your comments in the first round, and are glad that you have found the paper much improved.

Response to Reviewer #2

The authors did a very nice job responding to comments.

Thank you for your support and for your helpful feedback in the previous round.

Response to Reviewer #3

The authors have thoughtfully revised the manuscript and provided new information that help to clarify the work. Couple of further recommendations are as below -
In the new tables, it is not clear what the likelihood ratio test is comparing. The likelihood-ratio test assesses the goodness of fit of two competing statistical models based on the ratio of their likelihoods, so is each model significance relative to the baseline? Please clarify.

Thank you for your encouragement and your helpful comments. The likelihood ratio test is used to calculate statistical significance of the coefficients (and thereby the odds ratios). This is commonly done for generalized linear mixed models (1). We have edited the table legends to make this more clear.

(1) Barr, D. J. (2013). Random effects structure for testing interactions in linear mixed-effects models. Frontiers in Quantitative Psychology and Measurement, 328.

For Table 1b it would be good to reiterate that the OR is different than in Table 1a, i.e. in this case it is the OR with regards to adhering to the model's decision rather than the OR to call the police. Else it can be confusing. The observation that clinicians adhere significantly to the descriptive unbiased model choice but not to the biased model choice is puzzling. What explanation can the authors provide for the descriptive biased model not showing significance for clinicians?

Thanks for raising this point. In this case, we still estimate the OR with respect to the decision to call the police, and estimate the effect of an AI recommendation to call the police. Apologies for the confusion: we have now clarified this in the text and table.

You raise a good point on clinician non-adherence to a biased model with descriptive recommendations. We cannot be sure why exactly this is the case; however, we offer one potential explanation. On average, the biased model recommends police help more often than the unbiased model (see Supplementary Figure 1). Perhaps clinicians find it unreasonable to call the police as often as suggested by the biased model, though they broadly agree with the unbiased model. The fact that they override the model in the biased case further indicates that descriptive recommendations allow enough leeway for the clinicians to use their best judgement. We have added this explanation to the paper.
